# Detection of COPD exacerbations with continuous monitoring of breathing rate and inspiratory amplitude under oxygen therapy

**DOI:** 10.1186/s12911-025-02939-3

**Published:** 2025-02-25

**Authors:** Juliana Alves Pegoraro, Antoine Guerder, Thomas Similowski, Philippe Salamitou, Jesus Gonzalez-Bermejo, Etienne Birmelé

**Affiliations:** 1 Loewenstein Medical Technology GmbH+Co. KG, Karlsruhe, 76135 Germany; 2 AP-HP, Groupe Hospitalier Universitaire APHP-Sorbonne Université, Hôpital Pitié-Salpêtrière, Département R3S (Respiration, Réanimation, Réadaptation respiratoire, Sommeil), Service de médecine de readaptation respiratoire, Paris, F-75013 France; 3 INSERM, UMRS1158 Neurophysiologie Respiratoire Expérimentale et Clinique, Groupe Hospitalier Universitaire APHP-Sorbonne Université, site Pitié-Salpêtrière, Service de Réhabilitation Respiratoire, F-75013 Paris, France; 4 SRETT, 11 Rue Heinrich, 92100 Boulogne-Billancourt, France; 5 Institut de Recherche Mathématique Avancée, UMR 7501 Université de Strasbourg et CNRS, 7 rue René-Descartes, 67000 Strasbourg, France

**Keywords:** Respiratory pattern, Telemonitoring, Exacerbation detection, Classification, Novelty detection, Chronic obstructive pulmonary disease (COPD)

## Abstract

**Background:**

Chronic Obstructive Pulmonary Disease (COPD) is one of the main causes of morbidity and mortality worldwide. Its management represents real economic and public health burdens, accentuated by periods of acute disease deterioration, called exacerbations. Some researchers have studied the interest of monitoring patients’ breathing rate as an indicator of exacerbation, although achieving limited sensitivity and/or specificity. In this study, we look to improve the previously described method, by combining breathing variables, using multiple daily measures, and using an artificial intelligence-based novelty detection approach.

**Methods:**

Patients with COPD were monitored with a telemedicine device during their stay in a rehabilitation care center. Daily measures are compared to individually trained reference models based on: i. oxygen therapy duration ii. mean breathing rate, iii. mean inspiratory amplitude, iv. mean breathing rate and mean inspiratory amplitude, v. average distribution of breathing rate and inspiratory amplitude, vi. hidden Markov model (HMM) from a time series of breathing rate and inspiratory amplitude.

**Results:**

A set of 16 recordings with exacerbation and 23 recordings without exacerbation was obtained. When using a daily measure of breathing rate, pre-exacerbation periods were identified with a specificity of 50% and a sensitivity of 55.6%. The method based on daily oxygen therapy usage and the method based on time series obtain a sensitivity of 76.8% and 73.2%, respectively, for a fixed specificity of 50%.

**Conclusion:**

A single daily measure of breathing rate alone is not sufficient for the detection of pre-exacerbation periods. More complete models also achieve limited performance, equivalent to models based on changes in the duration of therapy usage.

**Supplementary Information:**

The online version contains supplementary material available at 10.1186/s12911-025-02939-3.

## Background

Chronic Obstructive Pulmonary disease (COPD) is a chronic disease mostly associated with tobacco smoking and characterized by chronic dyspnea, and repeated exacerbations. Although it is treatable and preventable at first stages, COPD is one of the main causes of morbidity and mortality worldwide, being a leading problem of public health [1].

During acute exacerbations of COPD, symptoms worsen and lung function declines. Prompt intervention is essential to mitigate the consequences, as delaying treatment is associated with poorer quality of life and greater risks of emergency hospital admissions [2]. Additionally, the mean cost for a hospitalisation due to an exacerbation can reach US$ 18,120 per year [3], further accentuating the economic burden of COPD.

Thereupon, ways of decreasing the cost of COPD management must be explored. Several researchers have considered the early detection of exacerbations through telemedecine and telemonitoring. The approaches are quite variable, concerning the exacerbation definition, the parameters monitored and the statistical methods used.

In some studies, telemonitoring is dependent on user intervention for measure acquisition, leading to inconsistent measurement frequency [4–6]. Meanwhile, monitoring devices coupled with non-pharmacological treatments are less dependent on manual measures and can still record relevant patient respiration data.

Non-invasive ventilation (NIV) devices, for example, can measure the breathing rate, percentage of triggered cycles, treatment adherence, unintentional leaks, and expired tidal volume [7–10]. However, although data is measured continuously during the treatment, studies usually retain only the central tendency and/or the dispersion of each indicator by day. Moreover, universal models and thresholds are often proposed, disregarding patients’ individuality and exacerbations variability, while still achieving promising results [10].

In the study of Borel and coll. [7], among the 64 patients included (with 21 exacerbations), the risk of exacerbation was increased when the breathing rate (sensitivity 46% and specificity 90%) was considered abnormally high for at least 2 days out of five consecutive days. For Blouet and coll. [8] (62 patients included and 21 patients with exacerbations), on the other hand, it was the variation of the breathing rate that achieved the best results. A model based on a standard deviation of breathing rate over 10 days resulted in a sensitivity of 62% and specificity of 76%.

In a different study with NIV data including 102 monitored patients (from which 31 were hospitalized), Jiang and coll. [9] predicted exacerbations based only on breathing rate with 93% sensitivity and 65% specificity. The model performance was increased when combining breathing rate with the number of days with abnormal values of daily use, leaks, and tidal volume.

Accordingly, NIV and long-term oxygen monitoring are now reimbursed in some countries such as France [11]. However, patients treated with NIV only correspond to a small portion of the population of COPD patients [12]. Therefore, more extensive monitoring tools must now be considered to cover a larger number of patients.

To our knowledge, the study of Yañez and coll. [13] is the only study that tries to predict exacerbations at home with long-term oxygen therapy data. In this study, 89 patients with COPD were included, from which 30 required hospitalization because of exacerbation. An increase in breathing rate was able to predict exacerbations with a sensitivity of 66% and specificity of 93%, 24 hours before hospitalization. Still, this study had many limitations, and the technology in 2012 was not ready for telemonitoring.

Thus, in this article, we look to confirm and improve these results by proposing new approaches for detecting periods of exacerbation. We hypothesize that, by combining breathing variables with new technology, using multiple daily measures, and using an artificial intelligence-based novelty detection approach, we will be able to detect a greater variety of pre-exacerbation periods.

## Methods

### Monitoring device

Patients were monitored using TeleOx^®^ (Srett, Boulogne-Billancourt, France). This device, placed on the oxygen circuit between the source and the nasal cannula of the patient, is designed to remote monitor therapy compliance. TeleOx^®^ also takes measures of the oxygen flow rate, breathing rate, and inspiratory amplitude of the patient every 5 minutes, allowing for frequent monitoring, with no new constraints for the patient. Additional information and image of the device can be found in Additional file 1.

### Data acquisition

Patients were enrolled while hospitalized at the rehabilitation care center of the *Groupe Hospitalier Pitié-Salpêtrière*, Sorbonne University, Paris, France. The unit receives COPD patients, who were previously hospitalized after an exacerbation, for about 6 weeks in a rehabilitation program.

The inclusion criteria required that patients had COPD, were under oxygen therapy, and were monitored with a TeleOx^®^. Only patients who could not provide informed consent were excluded. The protocol was reviewed and approved (CEPRO 2018-021) by the Institutional Review Board of the French learned society for respiratory medicine - *Société de Pneumologie de Langue Française*. Patients provided written informed consent.

In the week of admission, patients were equipped with one or two TeleOx^®^ devices according to the number of oxygen sources used by the patient. At the end of the stay, TeleOx^®^ data and the patient’s medical records were retrieved. The duration of stay depended on the success of the rehabilitation program or the need for a rehospitalization.

### Daily recordings descriptors

Every day, a TeleOx^®^ records 288 data points, containing the time, oxygen flow rate, median breathing rate, and median amplitude [14]. Only periods of acquisition corresponding to spontaneous breathing and with good signal quality are kept for analysis [15].

### Data labeling

In the novelty detection context, data is separated into baseline days (used to train the individual models) and test set days.

#### Baseline days

In the context of novelty detection, selecting appropriate baseline days is critical, as these days serve as reference for creating individual models. Therefore, baseline days should correspond to the patient’s stable state.

For each recording, the patient’s baseline is defined as the final week preceding medical discharge. Since data were only collected during the patient’s stay at the rehabilitation center, this final week is regarded as the most representative of the patient’s stable state within the available dataset.

According to the Mackay and coll. [16], it can take up to 12 days for a patient to return to baseline following an exacerbation. To ensure that stable days are appropriately used as baselines, we have established an arbitrary threshold of three weeks, retaining only recordings that extend beyond this duration for analysis.

#### Test days

The test set comprises days preceding an exacerbation and days of assumed stability. The exacerbation dates were defined by the physicians according to an increase in the symptoms (lasting at least 24 hours and detected with an EXASCORE greater than 3 [17] and confirmed by the physician) and leading to a change in medication.

Recordings containing one or more exacerbations are used to extract the pre-exacerbation days, which are the 4 (or less, if not available) days preceding an exacerbation.

Recordings containing no exacerbation compose the control group and are used to randomly extract 4 consecutive days of assumed stability, which are necessarily between the 15th day of recording and the first day of baseline. The resulting test set is then used to quantify the methods’ performances.

The process of sampling the test set is repeated 50 times, resulting in 50 ROC curves. The 50 ROC curves are used to estimate the mean ROC curve and its confidence interval for each considered method. The corresponding 50 areas under the curve (AUC) are used for method comparison using paired t-tests adjusted with the Benjamini and Yekutieli correction.

### Reference models

In previous studies, daily respiratory profiles were summarized by a single daily measure, computed as the central tendency of the measured points [7, 9, 13].

Hereafter, we compare the results using this approach with two other approaches: i. use the daily distribution of measures of breathing rate and amplitude and ii. use the complete time series of valid breathing rate and amplitude measures.

**Fig. 1:**
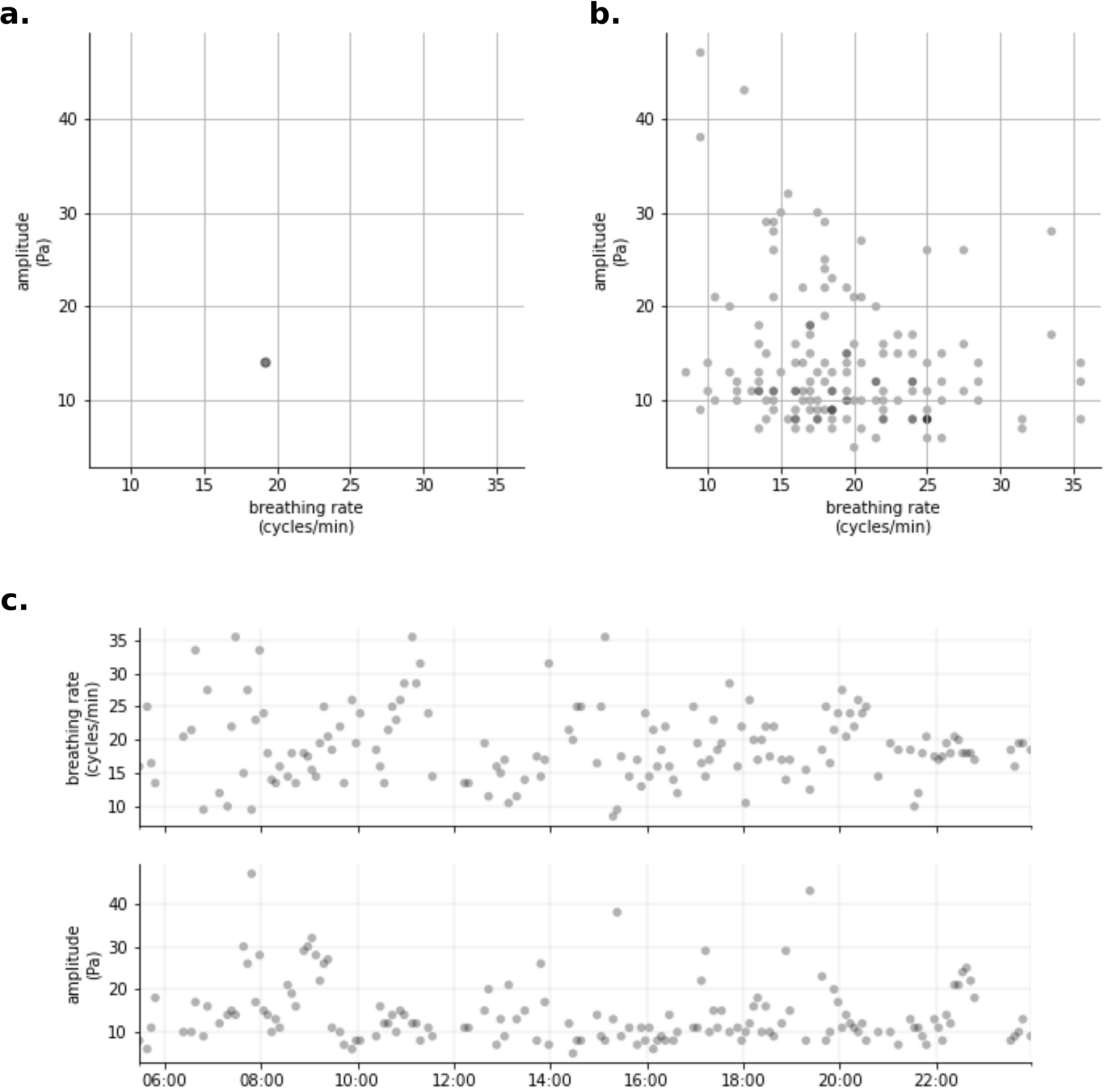
Data from the same day visualized according to three used approaches. **a** Single daily measure (mean) (**b**) Daily measures distribution (**c**) Daily time series

Figure 1 gives an example of daily data under each of the three used approaches. The Additional file 2 provides detailed information about the methods used.

First, the reference model is trained over the baseline week data for each recording. Then, a novelty score is computed for each day in the baseline week data. This allows for an estimation of the limits of normality, according to the mean and standard deviation of the novelty scores at baseline.

The novelty scores are then computed for each day in the test set. Based on these scores, the algorithm classifies each day as either “normal” or “atypical”, depending on whether its novelty score falls within the established limits of normality for that recording. Subsequently, periods of consecutive days are classified as either “stable” or “pre-exacerbation” according to the number of atypical days within the period. The performance of this classification approach is then compared across the different methods.

#### Day as a single measure

The simplest approach consists of considering that each day can be represented as a single measure, as previously used in other studies [7, 9, 13]. Figure 1a gives an example of daily data under this approach. In this example, the combination of mean daily breathing rate and mean daily inspiratory amplitude is presented. As previously described, the combination of breathing rate and amplitude allows for the detection of a greater number of respiratory mechanics adaptations, at least in short-term changes [15]. For comparison, the use of each variable alone is also tested.

With this approach, four baseline models are used, based on: daily oxygen therapy usage, mean of daily values of breathing rate alone, mean of daily values of inspiratory amplitude alone, and combination of mean breathing rate and mean amplitude.

The novelty score used with this approach is the Euclidean distance between the daily mean and the baseline week mean.

#### Day as a distribution

When each day is represented by the distribution of its measures, as in Fig. 1b, the baseline week can be described by the Wasserstein barycenter [18, 19] of the daily distributions. The barycenter corresponds to the distribution that is in the middle, that is to say, at the same distance from each of the days used as a reference.

With this approach, the novelty score is computed as the Wasserstein distance between the daily distribution and the baseline week barycenter.

#### Day as a time series

Recording data contains additional information about the order of the measures. For this reason, daily data can be visualized as a time series of breathing rate and amplitude (Fig. 1c). To take it into account, baseline week is modeled as a hidden Markov model (HMM) with two hidden states [20]. In a nutshell, the model considers that there are two hidden (not observed) states between which the patient transitions during the day according to estimated probabilities. The measures of breathing rate and inspiratory amplitude depend on the current hidden state and are described by Gaussian distributions. By combining those variables, the HMM can describe the normal behavior according to the observed series from the baseline week.

The novelty score in this approach is determined by the normalized score as used by Nishiyama and Shibata [21] and detailed in the Supplementary Material.

## Results

Recordings were retrieved between June 2019 and April 2021. In total 63 patients (39 male) were included, from which 6 had two or three periods of follow-up because of re-hospitalisation. This resulted in 70 recording periods described in Table 1. Twenty-nine recordings include at least one exacerbation, with 36 total exacerbations.

**Table 1:** Clinical characteristics of COPD patients at the beginning of monitoring

	Total ($\boldsymbol{N = 70}$)	With exacerbation ($\boldsymbol{N = 29}$)	Without exacerbation ($\boldsymbol{N = 41}$)
Men	42 (60.0%)	22 (75.9%)	20 (48.8%)
Age (years)	$69.0 \pm 7.8$	$69.3 \pm 7.9$	$68.8 \pm 7.8$
Height (cm)	$166.5 \pm 9.1$	$169.0 \pm 9.2$	$164.7 \pm 8.5$
Weight (kg)	$63.0 \pm 20.5$	$64.7 \pm 18.0$	$61.9 \pm 22.0$
FEV1 after bronchodilator (%)^a^	$34.3 \pm 16.4$ (24 missing^b^)	$34.0 \pm 16.5$ (11 missing^b^)	$34.5 \pm 16.3$ (13 missing^b^)
GOLD grade		GOLD 2: 1	GOLD 2: 1
		GOLD 3: 5	GOLD 3: 2
		GOLD 4: 14	GOLD 4: 26
		(9 missing^c^)	(12 missing^c^)
Initial 6MWT (%)^d^	$33.2 \pm 18.0$ (8 missing^e^)	$33.1 \pm 21.7$ (3 missing^e^)	$33.3 \pm 14.9$ (5 missing^e^)
Final 6MWT (%)^d^	$43.5 \pm 20.4$ (14 missing^e^)	$40.8 \pm 24.6$ (10 missing^e^)	$44.8 \pm 17.6$ (4 missing^e^)
Recording duration (days)	$31.5 \pm 15.7$	$29.1 \pm 13.8$	$33.2 \pm 16.7$

^a^ Forced expiratory volume in 1 second (FEV1) values were extracted from the spirometry examination performed closest to the recording period

^b^ For some patients, there was no spirometry result available in the medical record

^c^ For some patients, the information about GOLD grade was not found in the medical record

^d^ Results from the 6 minutes walking test (6MWT) as percentage of reference distance. The tests were performed in the first (Initial 6MWT) and last (Final 6MWT) weeks of monitoring

^e^ For some patients, the 6MWT was not performed in the first or last weeks of monitoring

Four recordings with exacerbation and 18 recordings without exacerbation last less than three weeks. From the remaining recordings containing an exacerbation, four present an exacerbation in the last days of follow-up. These recordings are excluded from the analysis since all weeks are considered inappropriate for the estimation of baseline. Another 5 recordings had data completely missing in the week preceding exacerbation and, for this reason, are also not included in the analysis.

**Fig. 2:**
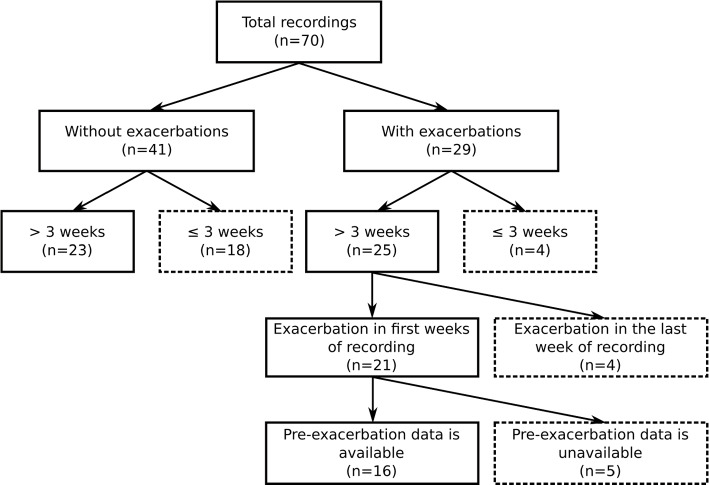
Schema of recordings in dataset. The recordings presented in a dashed box are not used in the analysis to classify a day as “normal” or “atypical”

The remaining data set contains 18 exacerbations from 16 recordings and 23 recordings without exacerbation. The recordings selection can be visualized in the schema in Fig. 2.

The distributions of mean breathing rate, mean inspiratory amplitude, and daily compliance across recordings with and without exacerbations is presented in Additional file 3.

**Fig. 3:**
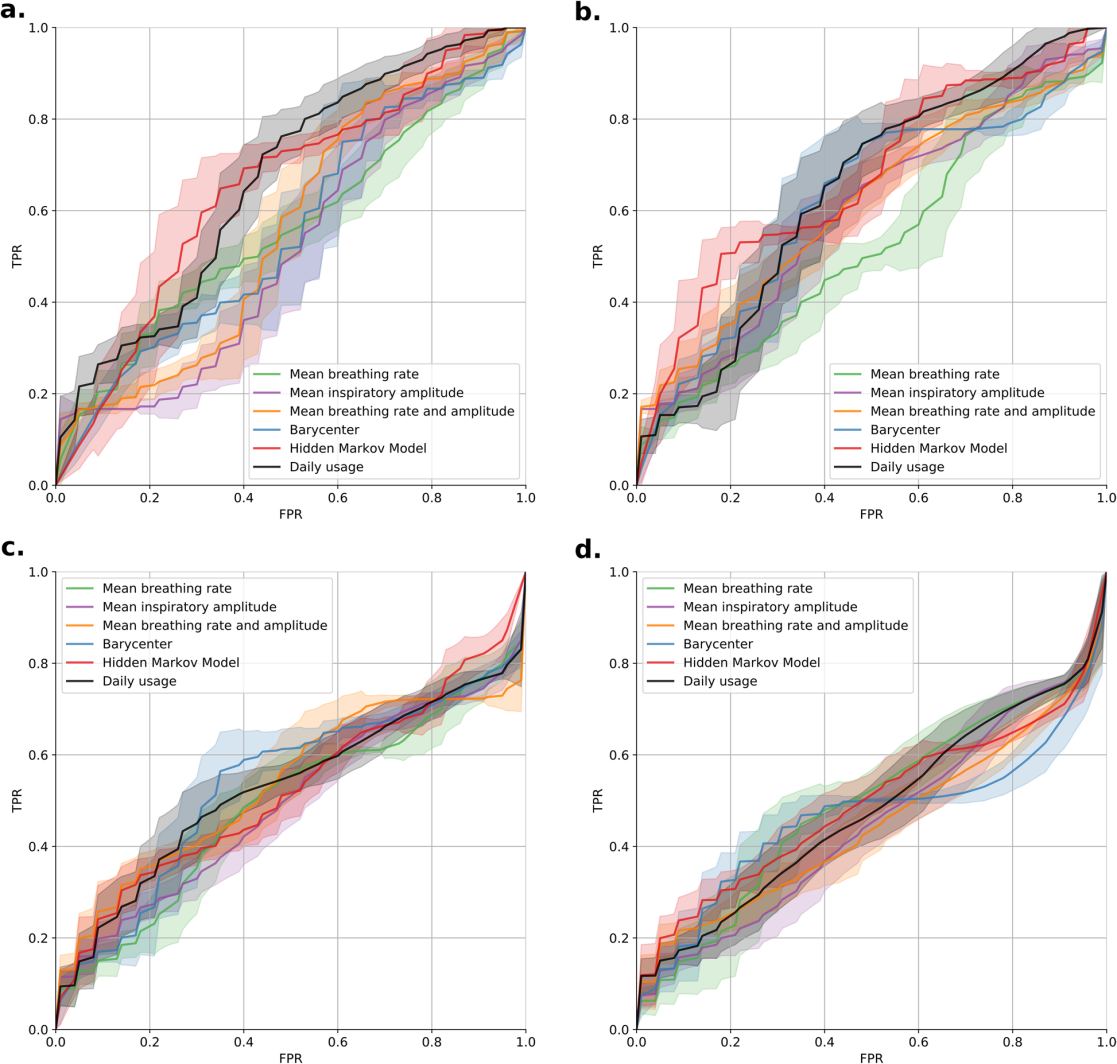
Performances of each method for the classification of pre-exacerbation periods when considering that a pre-exacerbation period is defined by at least *n* atypical days. **a** $n=1$ (**b**) $n=2$ (**c**) $n=3$ (**d**) $n=4$

The methods’ performances are estimated by considering that a period of 4 consecutive days is classified as pre-exacerbation if it includes at least a given number of atypical days. In Fig. 3, the ROC curves for each case are presented. Table 2 gives the corresponding performances concerning the average AUC and its standard deviation.

**Table 2:** AUC obtained for each tested method for identifying pre-exacerbation periods. AUC are presented as mean ± standard deviation

Method	Minimum atypical days in the tested period			
	1	2	3	4
Mean breathing rate	0.563 ± 0.035	0.527 ± 0.040	0.490 ± 0.033	0.492 ± 0.045
Mean inspiratory amplitude	0.520 ± 0.034	0.598 ± 0.033	0.494 ± 0.025	0.448 ± 0.038
Mean rate and amplitude	0.562 ± 0.030	0.611 ± 0.031	0.533 ± 0.026	0.448 ± 0.032
Wasserstein barycenter	0.554 ± 0.039	0.616 ± 0.033	0.535 ± 0.029	0.463 ± 0.027
Hidden Markov Model	0.650 ± 0.044	0.668 ± 0.029	0.523 ± 0.023	0.494 ± 0.025
Daily usage	0.664 ± 0.032	0.642 ± 0.045	0.526 ± 0.037	0.478 ± 0.042

The best results are achieved when considering that at least one or two days are atypical compared to baseline daily usage or baseline time series. These two methods obtain equivalent AUC (${P}=1.00$).

The average performances for the classification of stable and pre-exacerbation periods are compared to each of the other methods and the minimal number of atypical days using a one-sided paired t-test adjusted with the Benjamini and Yekutieli correction. The method based on the daily duration of oxygen therapy with $n=1$ and the method based on HMM with $n=2$ are equivalent (${P}=1.00$) and superior to the remaining methods tested (${P}<.001$).

For comparison, Table 3 gives the sensitivity obtained with each method when considering periods with at least one or two atypical days. For a specificity of 50%, the method based on HMM allows for the detection of on average 73% of the pre-exacerbation periods (accuracy of 62%), while the methods based on the daily mean breathing rate detect on average 56% (accuracy of 53%). The method based on the daily oxygen usage achieves 77% sensitivity with 63% accuracy.

**Table 3:** Sensitivities obtained with each method for a fixed specificity of 50

Method	Sentitivity	
	1 atypical day over 4	2 atypical days over 4
Mean breathing rate	55.6%	50.1%
Mean inspiratory amplitude	49.7%	66.4%
Mean breathing rate and amplitude	59.6%	66.1%
Wasserstein barycenter	51.8%	75.4%
Hidden Markov Model	73.2%	66.4%
Daily usage	76.8 %	75.5%

## Discussion

In this study, various models were applied for the identification of potential exacerbation events rooted in novelty detection methods. The novelty detection method is based on the assumption that these models can effectively characterize the typical state of an individual and that significant deviations from this baseline are particularly present in periods preceding exacerbations. Notably, the baseline models were described for each patient, granting a personalized representation of their stable state.

Upon analyzing the dataset, we found that relying solely on the mean breathing rate as a descriptor of the daily respiratory profile, as often presented in the literature [7, 9, 13], was not efficient in distinguishing stable and pre-exacerbation days. Specifically, using this approach with a fixed specificity of 50%, we observed that, on average, only 56% of exacerbations could be detected.

In contrast, a more complete method, based on time series and HMM, proved superior, detecting approximately 73% of exacerbations at the same level of false alerts. Notably, comparable performance was achieved by considering daily oxygen usage alone. Despite the similar outcomes, the latter approach offers simplicity in estimation. This observation highlights the importance of monitoring a patient’s adherence to therapy.

It is of note that the performances observed in previously published studies are mainly higher than those obtained in our study. The current data have been collected from patients at the rehabilitation care center. Patients who are admitted to the unit have recently experienced an exacerbation requiring hospitalization and have not yet recovered enough to return home. Still, it is expected that their health status will improve during their stay, approaching their “baseline” before hospital discharge. This leads to two major difficulties in the study of the dataset. First, patients are not actually at their stable state in the defined baseline periods. Instead, they are improving toward stability, and the chosen baseline days might be too close to the previous exacerbation event. Second, among the patients who don’t experience an exacerbation during the recording period, changes in the respiratory profile are also present, but related to a positive outcome, a consequence of the rehabilitation process. Thus, in this context, it is difficult to efficiently separate recovery from exacerbation. In this sense, the methods tested should be more useful when applied to stable patients. On the other hand, the close supervision by the medical staff in the context of hospitalized patients allows for a clearer definition of the exacerbation onset, which might be more challenging in studies including patients at home.

Contrastingly, most previous studies chose to follow patients at home and for longer periods (at least 3 months) [7–9, 13]. In the case of the study conducted by Yañez and coll. [13], only patients initially in a stable state (at least 8 weeks since the last change in treatment or hospitalization) were included, enabling a more accurate description of the patient’s baseline. Additionally, Yañez and Coll. [13] analyze only patients with exacerbations, leading to a high prevalence of exacerbations and inducing higher sensitivity and specificity. Finally, automatic remote monitoring was not feasible in the study, because this version of the device was not communicating, but a communicating version is available.

Despite the described dataset limitations, this study presents some alternative methods for a more complete monitoring of patient’s respiratory profile changes. It starts with the detailed monitoring of the patient’s features with TeleOx^®^, and it follows with the proposed method that models the baseline period with an HMM based on the observations of breathing rate and inspiratory amplitude as time series.

Accordingly, further work should focus on a real-life context, with the remote monitoring of patients at home and over a longer period. The new reimbursement aboit monitoring patients under long term oxygenotherapy will allow such studies and results. By limiting the interference of very recent exacerbations, it should be possible to confirm the interest of the method based on HMM and daily time series of breathing rate and inspiratory amplitude in comparison to the monitoring of therapy adherence alone.

## Conclusion

In conclusion, the monitoring device not only enables remote monitoring of therapy adherence but also delivers frequent measurements of key physiological indicators, including breathing rate and inspiratory amplitude. Our study highlights the challenge of detecting pre-exacerbation periods, pointing out that this task exceeds the capabilities of relying solely on a single daily breathing rate measure. More complete models integrating artificial intelligence with time series measurements and additional physiological parameters, such as amplitude and hours of utilization, show promise for improved performance. Currently, AI is not ready to replace human follow-up care, and continued advancements are essential to enhance healthcare technologies and their integration into clinical practice.

## Supplementary Information

Supplementary Material 1.

Supplementary Material 2.

Supplementary Material 3.

## Data Availability

The datasets used and/or analyzed during the current study are available from the corresponding author on reasonable request.

## Abbreviations

6MWT: 6 minutes walking test
AUC: Area under the curve
COPD: Chronic obstructive pulmonary disease
FEV1: Forced expiratory volume in 1 second
HMM: Hidden Markov model
NIV: Non-invasive ventilation
