# Supplementary material for: Detection of COPD exacerbations with continuous monitoring of breathing rate and inspiratory amplitude under oxygen therapy
Source: BMC Med Inform Decis Mak. 2025 Feb 25;25:101. doi: 10.1186/s12911-025-02939-3 (PMC11863910; doi:10.1186/s12911-025-02939-3)
Supplement: Supplementary file 1 — Supplementary Material 1. [file 12911_2025_2939_MOESM1_ESM.pdf]

Additional file 1 - TeleOx®

TeleOx® (Srett, Boulogne-Billancourt, France) is a medical device designed to evaluate adherence and treatment efficacy in long-term oxygen therapy (LTOT) patients (Figure 1). The device is placed on the oxygen circuit between the source and the nasal cannula of the patient (Figure 2), adding no new constraints for the patient. It allows for a continuous follow-up, with no invasive device nor manipulation needed.

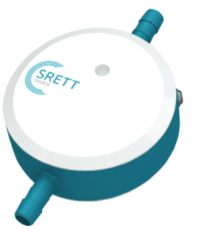

Figure 1: Photo of TeleOx®

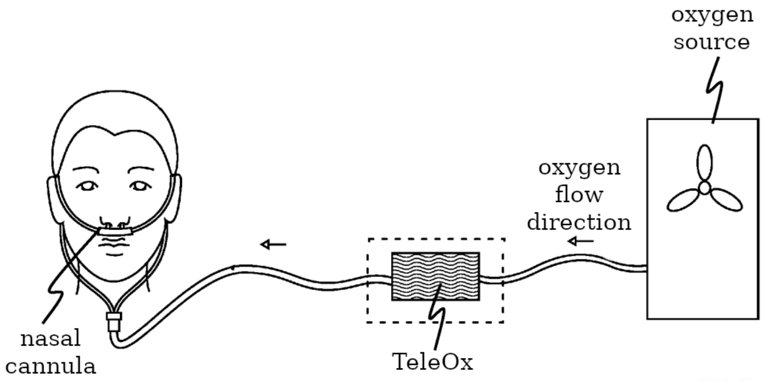

Figure 2: Oxygen circuit with TeleOx® [1]

References

[1] Philippe Salamitou and Xuan Loc Le. *Oxygen therapy monitoring device and method*. U.S. Patent App. 15/760,102. Sept. 2018.
