# Supplementary material for: Detection of COPD exacerbations with continuous monitoring of breathing rate and inspiratory amplitude under oxygen therapy
Source: BMC Med Inform Decis Mak. 2025 Feb 25;25:101. doi: 10.1186/s12911-025-02939-3 (PMC11863910; doi:10.1186/s12911-025-02939-3)
Supplement: Supplementary file 2 — Supplementary Material 2. [file 12911_2025_2939_MOESM2_ESM.pdf]

Additional file 2 - Methods

In this paper, we propose modeling the patient's respiratory profile based on i. use the daily distribution of measures in the plan breathing rate-amplitude and ii. use the complete time series of valid breathing rate and amplitude measures.

In the first case, the method used consists of modeling the baseline week as a Wasserstein barycenter and using the Wasserstein distance from the reference model as a novelty score. In the second approach, the baseline week is modeled by a Hidden Markov Model and the log-likelihood under the model is used as novelty score.

Hereafter, the proposed methods are described.

Wasserstein distance

The distance between two distributions can be quantified using optimal transport theory. The idea behind it can be explained with the pile of sand analogy. We consider an initial distribution of mass μ that describes the pile of sand. We wish to move each grain of sand so the final distribution is ν. The ground cost for transporting one grain of sand from position x to position y is defined by the cost function $c(x, y)$. The optimal distance between the piles is the distance that minimizes the overall cost of transportation [3, 4]. The Wasserstein distance is this optimal value, that quantifies the similarity between two distributions.

For the comparison of sets of points, discrete distributions are defined according to the points weights and locations. Equation 1 defines the discrete distribution μ, according to the weights α and locations of points $x_1, \ldots, x_n$, where δ_x is the Dirac at position x.

$$\mu = \sum_{i=1}^{n} \alpha_i \delta_{x_i} \tag{1}$$

In the Kantorovich formulation for discrete distributions, the distance between μ and ν can be estimated according to Equation 2, where Π are the admissible transport plans between μ and ν. The transport plan can be understood as the matrix that defines the amount of sand π_{ij} that is to be transported from x_i to y_j. Thus, the mass moved from each distribution corresponds to its initial mass ($\sum_{j=1}^{m} \pi_{ij} = \mu_i$ and $\sum_{i=1}^{n} \pi_{ij} = \nu_j$) and the mass displaced is always positive ($\pi_{ij} \geq 0$). The total transportation cost is then

$$W(\mu, \nu) = \min_{\pi \in \Pi} \sum_{i=1}^{n} \sum_{j=1}^{m} \pi_{ij} c(x_i, y_j) \tag{2}$$

As μ and ν are distributions, and all measures of a given day are considered of equal importance, we use that all weights within a day containing n data point are $\alpha_i = 1/n$. Moreover, to ensure that breathing rate and amplitude have the same impact, the cost function used is the Mahalanobis distance.

Wasserstein barycenters

Based on the Wasserstein distance between discrete distributions, the Wasserstein barycenter is a weighted average distribution ν that is at midway from a given set of distributions $\{\mu_j\}$. The Wasserstein barycenter of the distributions $\{\mu_j\}$ is estimated with Equation 3, where the weights $\rho_j \geq 0$ and $\sum_j \rho_j = 1$.

$$\nu = \min_{\nu} \sum \rho_j W(\nu, \mu_j) \tag{3}$$

In the reference models, we give uniform weights to all reference days and we estimate Wasserstein barycenters with 288 points, independently of the number of valid measures available in the original daily distributions. This corresponds to a complete day of oxygen therapy adherence under TeleOx® monitoring, where a valid measure is recorded every 5 minutes.

Hidden Markov Model

A Markov model is a stochastic model that describes a Markov process. A Markov process is a chain of events where the probability of the event at time $t+1$ depends only on the event at time t, and not on previous events (on $t-1, t-2, \ldots$). This assumption is defined in Equation 4 for a sequence of states $s_0, s_1, \ldots, s_T$.

$$P(s_t = i | s_0, \ldots, s_{t-1}) = P(s_t = i | s_{t-1}) \tag{4}$$

A representation of a Markov chain with two states i and j is given in Figure 1. The series $S = s_0, s_1, \ldots, s_T$ is a series of states that can get values i or j. The Markov chain is described by the parameters $\Pi_i = P(s_0 = i)$, the initial state probability, and $A = \{a_{ij}\} = P(s_t = j | s_{t-1} = i)$, the transition probability matrix, which is independent of time.

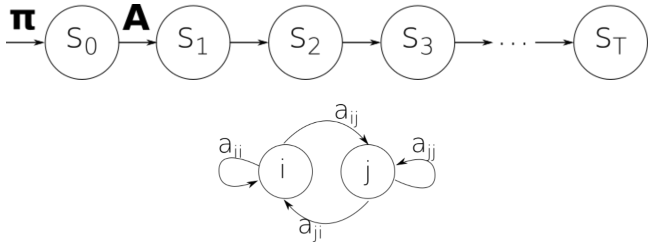

Figure 1: Markov chain diagrams for two observable states

In the HMM the series of states $S = s_0, s_1, \ldots, s_T$ is not directly observed. Instead, the observations $O = o_0, o_1, \ldots, o_T$ are variables that depend on the hidden state. Thus, a new parameter $B = \{b_j(o_t)\} = P(o_t | s_t = j)$ defines the emission probabilities.

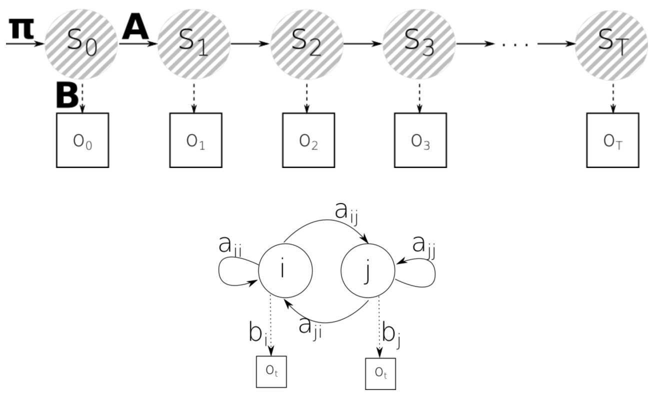

Figure 2: Hidden Markov model diagrams for two hidden states

Estimate the model parameters

A hidden Markov model is described by the parameter $\Theta = (A, B, \Pi)$. Θ can be estimated from the observed data using the Baum-Welch algorithm, which is a special case of the expectation-maximization (EM) algorithm [1].

The objective of the algorithm is to find the local maximum for $\Theta^* = \Theta \text{argmax}\,(O|\Theta)$ through iterations of the forward and backward steps.

Θ is initialized randomly or with prior information. The algorithm starts with the forward procedure that consists of estimating $\alpha_t(j) = P(o_0, o_1, \ldots, o_t, s_t = j|\Theta)$, the probability of observing $O_{0:t}$ and being at state j at time t. The computation is recursive, with the following equations:

$$\text{Initialization:} \qquad \alpha_0(i) = \pi_i \times b_i(o_0)$$

$$\text{Recursion:} \qquad \alpha_{t+1}(i) = b_i(o_{t+1}) \sum_j^N \alpha_t(j) a_{ji}, \text{ with } 1 \leq j \leq N, 0 \leq t \leq T$$

Next, the backward procedure is used to estimate $\beta_t(j) = P(o_{t+1}, \ldots, o_T|s_t = j, \Theta)$, the probability of future observations $O_{t+1:T}$ given that the state is j at time t. Recursive computation uses the following equations:

$$\text{Initialization:} \qquad \beta_T(i) = 1$$

$$\text{Recursion:} \qquad \beta_t(i) = \sum_j^N \beta_{t+1}(j) a_{ij} b_j(o_{t+1}), \text{ with } 1 \leq j \leq N, 0 \leq t \leq T$$

Then, two temporary variables are estimated. The state occupation probability $\gamma_t(i) = P(s_t = i|O, \Theta)$ is the probability of occupying state i at time t given the series of observations O and the parameters Θ. $\xi_t(i,j) = P(s_t =$

$i, s_{t+1} = j|O, \Theta)$ is the probability of occupying state i at time t and state j at time $t+1$ given the observation O and the parameters Θ.

$$\gamma_t(i) = \frac{P(O, s_t = i|\Theta)}{P(O|\Theta)} = \frac{\alpha_t(i)\beta_t(i)}{\sum_j^N \alpha_t(j)\beta_t(j)}$$

$$\xi_t(i,j) = \frac{P(s_t = i, s_{t+1} = j, O|\Theta)}{P(O|\Theta)} = \frac{\alpha_t(i) a_{ij} \beta_t(j) b_j(o_{t+1})}{\sum_t^N \sum_w^N \alpha_t(k) a_{kw} \beta_{t+1}(w) b_w(p_{t+1})}$$

Finally, the parameters are updated using:

$$\pi_i^* = \gamma_0(1)$$

$$a_{ij}^* = \frac{\sum_{t=1}^{T-1} \xi_t(i,j)}{\sum_{t=1}^{T-1} \gamma_t(i)}$$

$$b_i^*(v_k) = \frac{\sum_{t=1}^{T} 1_{o_t=v_k} \gamma_t(i)}{\sum_{t=1}^{T} \gamma_t(i)}, \text{ with } 1_{o_t=v_k} = \begin{cases} 1 & \text{if } o_t = v_k \\ 0 & \text{otherwise} \end{cases}$$

For the estimation of the respiratory profile models, each day is considered as a sequence of observations O_r. The model is fitted to R sequences $O_1, \ldots, O_R$ by estimating temporary variables $\gamma_{r,t}(i)$ and $\xi_{r,t}(i,j)$ for each sequence of observations. The parameters π, A and B are updated according to the following equations :

$$\pi_i^* = \frac{\sum_{r=1}^{R} \gamma_0(1)}{R}$$

$$a_{ij}^* = \frac{\sum_{r=1}^{R} \sum_{t=1}^{T-1} \xi_{r,t}(i,j)}{\sum_{r=1}^{R} \sum_{t=1}^{T-1} \gamma_{r,t}(i)}$$

$$b_i^*(v_k) = \frac{\sum_{r=1}^{R} \sum_{t=1}^{T} 1_{o_{r,t}=v_k} \gamma_{r,t}(i)}{\sum_{r=1}^{R} \sum_{t=1}^{T} \gamma_{r,t}(i)}, \text{ with } 1_{o_t=v_k} \begin{cases} 1 & \text{if } o_t = v_k \\ 0 & \text{otherwise} \end{cases}$$

These steps are repeated iteratively until convergence, the reference model is thus defined by the parameter $\Theta = (A, B, \Pi)$.

Series likelihood under HMM

The likelihood of a sequence of observations given the model parameters can be estimated using the Forward algorithm. Instead of considering all marginal conditions, the Forward algorithm considers the conditional independence rule of the HMM that states that the observation o_t only depends on the state s_t and that the current state s_t only depends on the previous state s_{t-1}.

Thus, the probability of the observation o_t being in state i ($\alpha_t(i)$) can be estimated according to the probabilities associated with the previous observations $o_{0:t}$. The recursive computation of the likelihood of the sequence of observations corresponds to the forward procedure that was used to estimate the model parameters.

Score normalization for the method based on HMM

The likelihood of a sequence of observations O given the model parameters Θ is influenced by the length of the sequence. Thus, the daily scores are normalized using the *self model*, Θ_O, that is HMM generated from the daily sequence of observations O.

The normalized daily score is given by $P^*(O|\Theta_M) = \frac{P(O|\Theta_M)}{P(O|\Theta_O)}$ [2], where $P(O|\Theta_M)$ and $P(O|\Theta_O)$ are the likelihoods of the daily sequence O given the patient's baseline model Θ_M and the *self model* Θ_O.

References

[1] Charu C Aggarwal. *Outlier Analysis*. 2nd ed. New York, USA: Springer, 2017.

[2] Mio Nishiyama and Tadashi Shibata. "Normalized scoring of hidden Markov models by on-line learning and its application to gesture-sequence perception". In: *Proceedings of the 16th IEEE International Conference on Image Processing*. ICIP'09. Cairo, Egypt: IEEE Press, 2009, pp. 3529–3532. ISBN: 9781424456536.

[3] Gabriel Peyré, Marco Cuturi, et al. "Computational optimal transport: With applications to data science". In: *Foundations and Trends in Machine Learning* 11.5-6 (2019), pp. 355–607.

[4] Julien Rabin et al. "Wasserstein barycenter and its application to texture mixing". In: *International Conference on Scale Space and Variational Methods in Computer Vision*. Springer. 2011, pp. 435–446.
