# Supplementary material for: Detection of COPD exacerbations with continuous monitoring of breathing rate and inspiratory amplitude under oxygen therapy
Source: BMC Med Inform Decis Mak. 2025 Feb 25;25:101. doi: 10.1186/s12911-025-02939-3 (PMC11863910; doi:10.1186/s12911-025-02939-3)
Supplement: Supplementary file 3 — Supplementary Material 3. [file 12911_2025_2939_MOESM3_ESM.pdf]

Additional file 3 - Distributions per group

Figure 1 shows the distribution of mean breathing rate, mean inspiratory amplitude, and daily compliance across two groups of recordings: those with and those without exacerbations. For recordings with exacerbations, distributions are shown for both baseline and pre-exacerbation periods. For recordings without exacerbations, distributions are provided for baseline and test days.

As described in the Methods section, our primary analysis used 50 iterations to randomly select sets of 4 consecutive test days to assess the methods' performance. In Figure S1, all test days are combined and displayed as a single distribution.

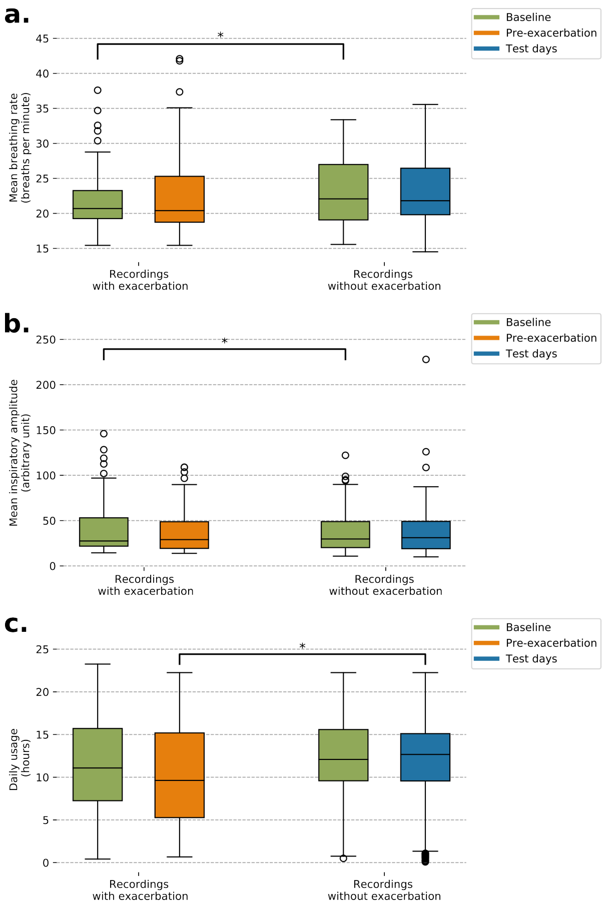

Figure 1: Distribution of daily parameters **a.** mean breathing rate, **b.** mean inspiratory amplitude and **c.** daily usage in periods of baseline, pre-exacerbation, and test days without exacerbation. The asterisk indicates statistically significant differences ($p < 0.05$).
